# Supplementary material for: The Hidden Landscape of Missed Effects in Human Functional Neuroimaging
Source: bioRxiv. 2026 May 24:2026.05.21.726948. Preprint. [Version 1] doi: 10.64898/2026.05.21.726948 (PMC13228250; doi:10.64898/2026.05.21.726948)
Supplement: Supplement 2 [file media-2.pdf]

Supplementary Tables & Figures

Study Name (dataset_map_test_task_variables)	Dataset	N	In-Scanner Task	Outcome Measure	Measure Info
abcd_fc_r_REST_age_months	abcd	8326	REST	age_months	Age in months
abcd_fc_r_REST_bmi	abcd	8320	REST	bmi	Body Mass Index
abcd_fc_r_REST_cbcl_scr_syn_aggressive_t_FU1	abcd	7899	REST	cbcl_scr_syn_aggressive_t_FU1	Aggressive CBCL Syndrome Scale (t-score) at follow-up 1
abcd_fc_r_REST_cbcl_scr_syn_aggressive_t	abcd	8362	REST	cbcl_scr_syn_aggressive_t	Aggressive CBCL Syndrome Scale (t-score)
abcd_fc_r_REST_cbcl_scr_syn_anxdep_t_FU1	abcd	7899	REST	cbcl_scr_syn_anxdep_t_FU1	AnxDep CBCL Syndrome Scale (t-score)
abcd_fc_r_REST_cbcl_scr_syn_attention_t_FU1	abcd	7899	REST	cbcl_scr_syn_attention_t_FU1	Attention CBCL Syndrome Scale (t-score)
abcd_fc_r_REST_cbcl_scr_syn_attention_t	abcd	8362	REST	cbcl_scr_syn_attention_t	Attention CBCL Syndrome Scale (t-score)
abcd_fc_r_REST_cbcl_scr_syn_external_t_FU1	abcd	7899	REST	cbcl_scr_syn_external_t_FU1	External CBCL Syndrome Scale (t-score)
abcd_fc_r_REST_cbcl_scr_syn_external_t	abcd	8362	REST	cbcl_scr_syn_external_t	External CBCL Syndrome Scale (t-score)
abcd_fc_r_REST_cbcl_scr_syn_internal_t_FU1	abcd	7899	REST	cbcl_scr_syn_internal_t_FU1	Internal CBCL Syndrome Scale (t-score)
abcd_fc_r_REST_cbcl_scr_syn_internal_t	abcd	8362	REST	cbcl_scr_syn_internal_t	Internal CBCL Syndrome Scale (t-score)
abcd_fc_r_REST_cbcl_scr_syn_rulebreak_t_FU1	abcd	7899	REST	cbcl_scr_syn_rulebreak_t_FU1	RuleBreak CBCL Syndrome Scale (t-score)
abcd_fc_r_REST_cbcl_scr_syn_rulebreak_t	abcd	8362	REST	cbcl_scr_syn_rulebreak_t	RuleBreak CBCL Syndrome Scale (t-score)
abcd_fc_r_REST_cbcl_scr_syn_social_t_FU1	abcd	7899	REST	cbcl_scr_syn_social_t_FU1	Social CBCL Syndrome Scale (t-score) at follow-up 1
abcd_fc_r_REST_cbcl_scr_syn_somatic_t_FU1	abcd	7899	REST	cbcl_scr_syn_somatic_t_FU1	Somatic CBCL Syndrome Scale (t-score)
abcd_fc_r_REST_cbcl_scr_syn_thought_t_FU1	abcd	7899	REST	cbcl_scr_syn_thought_t_FU1	Thought CBCL Syndrome Scale (t-score)
abcd_fc_r_REST_cbcl_scr_syn_thought_t	abcd	8362	REST	cbcl_scr_syn_thought_t	Thought CBCL Syndrome Scale (t-score)
abcd_fc_r_REST_cbcl_scr_syn_withdep_t_FU1	abcd	7899	REST	cbcl_scr_syn_withdep_t_FU1	WithDep CBCL Syndrome Scale (t-score)
abcd_fc_r_REST_nihtbx_fluidcomp_fc	abcd	7713	REST	nihtbx_fluidcomp_fc	Cognition Fluid Composite Fully-Corrected T-score
abcd_fc_r_rest_pea_wiscv_tss	abcd	7628	REST	pea_wiscv_tss	WISC-V Matrix Reasoning Total Scaled Score
abcd_fc_t_MID_REST	abcd	7496	MID, REST	task - rest	
abcd_fc_t_nBack_REST	abcd	7360	nBack, REST	task - rest	
abcd_fc_t_SST_REST	abcd	7417	SST, REST	task - rest	
abcd_fc_t2_REST_sex	abcd	8363	REST	sex	
hbn_fc_r_rest_nih_card	hbn	449	REST	card	Dimensional Change Card Sort Age 3+ Age-adjusted Scale Score
hbn_fc_r_rest_nih_flanker	hbn	449	REST	flanker	Flanker Inhibitory Control and Attention Age 3+ Age-adjusted Score

hbn_fc_r_rest_nih_list	hbn	449	REST	list	List Sorting Working Memory Age 7+ Age-adjusted Score
hbn_fc_r_rest_nih_process	hbn	449	REST	process	Pattern Comparison Process Speed 7+ Age-adjusted Score
hbn_fc_r_rest_srs_srs	hbn	641	REST	srs	Social Responsiveness Scale
hbn_fc_t2_rest_diag_diag	hbn	445	REST	diag	Diagnosis
hcp_act_t_EMOTION	hcp	314	EMOTION	task activation	
hcp_act_t_GAMBLING	hcp	324	GAMBLING	task activation	
hcp_act_t_RELATIONAL	hcp	314	RELATION AL	task activation	
hcp_act_t_SOCIAL	hcp	317	SOCIAL	task activation	
hcp_act_t_WM	hcp	324	WM	task activation	
hcp_fc_r_REST_PMAT24_A_CR	hcp	1060	REST	PMAT24_A_CR	Fluid Intelligence (Penn Progressive Matrices): number of correct responses
hcp_fc_r_REST_ProcSpeed_AgeAdj	hcp	1067	REST	ProcSpeed_AgeAdj	Processing Speed (Pattern Completion Processing Speed): age adjusted scale score
hcp_fc_t_REST_EMOTION	hcp	1023	EMOTION, REST	task - rest	HCP Emotion task
hcp_fc_t_REST_GAMBLING	hcp	1058	GAMBLING, REST	task - rest	HCP Gambling task
hcp_fc_t_REST_RELATIONAL	hcp	1017	RELATION AL, REST	task - rest	HCP Relational task
hcp_fc_t_REST_SOCIAL	hcp	1028	SOCIAL, REST	task - rest	HCP Social task
hcp_fc_t_REST_WM	hcp	1059	WM, REST	task - rest	HCP Working memory task
hcp_fc_t2_REST_Gender	hcp	1067	REST, REST	Gender	
pnc_fc_r_REST_ADD011	pnc	1248	REST	ADD011	Attention Deficit Disorder: Did you often have trouble paying attention or keeping your mind on your school, work, chores, or other activities that you were doing?
pnc_fc_r_REST_DEP001	pnc	1251	REST	DEP001	Depression: Has there ever been a time when you felt sad or depressed most of the time?
pnc_fc_r_REST_EAT001	pnc	1248	REST	EAT001	Eating Disorder: Was there ever a time when you felt really fat or heavy, but other people said that you were too thin?
pnc_fc_r_REST_GAD001	pnc	1248	REST	GAD001	Generalized Anxiety Disorder: Have you ever been a worrier?
pnc_fc_r_REST_MAN001	pnc	1262	REST	MAN001	Mania/ Hypomania: Have there been times when you were much more active, excited or energetic than usual, had problems sitting still, or needed to move around a lot?

pnc_fc_r_REST_OCD018	pnc	1262	REST	OCD018	Obsessive Compulsive Disorder: Have you ever saved up so many things that people complained or they got in the way?
pnc_fc_r_REST_PAN001	pnc	1252	REST	PAN001	Panic Disorder: Have you ever had an attack like this?
pnc_fc_r_REST_PSY001	pnc	1246	REST	PSY001	Psychosis: Have you ever heard voices when no one was there?
pnc_fc_r_REST_SIP003	pnc	1247	REST	SIP003	SIPS - PRIME SCREEN-REVISED: I think that I have felt that there are odd or unusual things going on that I can't explain.
pnc_fc_t2_REST_Sex	pnc	1267	REST	Sex	Sex of participant. 0: unknown, 1: female, 2: male
slim_fc_r_REST_State_Anxiety	slim	473	REST	State_Anxiety	State anxiety measured with the State Trait Anxiety Inventory (STAI)
slim_fc_t2_REST_Sex	slim	544	REST	Sex	Participant sex
ukb_fc_r_rest_age	ukb	40925	REST	age	Age in years
ukb_fc_r_rest_fluid_intelligence	ukb	37730	REST	fluid_intelligence	Fluid intelligence [Field ID: 20016]: verbal and numerical reasoning multiple-choice questions answered correctly in two minutes (total=13).
ukb_fc_t2_rest_gender	ukb	40925	REST	gender	Participant gender

SI Table 1. Dataset characteristics for each study. Dataset, sample size, neuroimaging measures, and outcome measures are provided for each study. Outcome measures encompass demographic variables, cognitive assessments, clinically relevant assessments, psychiatric screening questions, and task performance metrics across multiple cognitive domains.

Dataset	Task	Task Info	Scan Duration
abcd	REST	Eyes open. Participants were instructed to relax while maintaining passive visual fixation on a crosshair presented centrally on screen.	20 minutes total, with only 10 minutes used by the contributor for FC calculations
abcd	MID	Participants view incentive cues indicating potential monetary outcomes (Win \$0.20, Win \$5.00, Lose \$0.20, Lose \$5.00, or neutral \$0), followed by a variable delay period, then a brief target stimulus (150-500ms) requiring a speeded button press response. Must always respond regardless of trial type with fast responses on win trials to earn money, fast responses on loss trials to avoid losing money, while slow responses result in no monetary gain or loss of money, respectively. Task difficulty adjusted to maintain approximately 60% success rate across all participants.	20 minutes rest scan with only 10 minutes used by the contributor for FC calculation and 5.7 minutes task scan
abcd	nBack	The Emotional N-back (EN-back) task is a working memory paradigm that measures both cognitive control and emotion regulation. The task consists of two runs with eight blocks each, where participants view sequences of happy faces, fearful faces, neutral faces, and places for 2 seconds per stimulus, followed by 500ms fixation periods. In the 0-back condition (low memory load), participants respond "match" when the current stimulus matches a target image shown at the beginning of the block, essentially testing simple recognition. In the 2-back condition (high memory load), participants must respond "match" when the current stimulus is identical to the one presented two trials earlier. Each block contains 10 trials with 2 targets, 2-3 non-target lures, and several non-lures, totaling 160 trials across both runs with 80 trials per memory condition and 40 trials per stimulus type. [1,2]	20 minutes rest scan with only 10 minutes used by the contributor for FC calculation and 5.7 minutes task scan

abcd	SST	Participants respond to directional arrows (left/right) as quickly as possible on "Go" trials, but must withhold their response when an unpredictable "Stop" signal (upward arrow) appears. Each of 2 runs contains 180 trials (150 Go trials, 30 Stop trials) with each trial lasting 1000ms. A tracking algorithm adjusts the Stop Signal Delay (SSD) to maintain approximately 50% successful inhibitions. SSD decreases time by 50ms after failed stops (easier) and increases by 50ms after successful stops (harder). Each run lasts 349 seconds total.	20 minutes rest scan with only 10 minutes used by the contributor for FC calculation and 5.7 minutes task scan
hbn	REST	Exact quote: The participant was presented a white fixation cross in the center of a black screen and instructed to rest with eyes open. Specific instructions were as follows: "Please lie quietly with your eyes open, and direct your gaze towards the plus symbol. During this scan let your mind wander. If you notice yourself focusing on a particular stream of thoughts, let your mind wander away." (O'Connor et al. 2017)	Initially 10 minutes resting state scan eventually broken into two 5 minute scans. In both cases, all 10 minutes were used for FC calculations by the contributor
hcp	EMOTIO N	Subjects must decide which of two faces, with either fearful or angry expressions, presented on the bottom of the screen, matches the face at the top of the screen, or which of two shapes presented at the bottom of the screen matches the shape at the top of the screen. The contrast used in the analysis is comparing activation during face-matching blocks vs shape-matching blocks.	Each run contains 6 blocks (3 face + 3 shape) at 21 seconds per block plus 8 seconds of final fixation, totaling 134 seconds (~2.2 minutes) per run, with 2 runs making the entire emotion task approximately 4.5 minutes long.
hcp	GAMBLI NG	Participants play a card guessing game where they predict whether a mystery card number (1-9) is higher or lower than 5, receiving feedback in the form of monetary rewards (green arrow, +$1), losses (red arrow, -$0.50), or neutral outcomes (gray arrow, number 5). Each trial consists of a decision phase (up to 1500 ms), feedback (1000 ms), and fixation (1000 ms). The contrast is biased toward mostly rewards vs biased towards mostly losses. Each of two runs contains 2 reward blocks, 2 loss blocks, and 4 fixation blocks (15 seconds each).	Each run contains 4 task blocks (8 trials each at 3.5 seconds per trial) plus 4 fixation blocks (15 seconds each), totaling approximately 2.9 minutes per run, with 2 runs making the entire gambling task about 5.8 minutes long.
hcp	RELATIO NAL	Participants view shapes with different textures and perform two types of tasks: identifying whether two pairs of objects differ along shape or texture, and determining if a bottom object matches top objects on a specified shape or texture indicated by a word cue. The relational condition uses 4 trials per block (3500 ms stimulus, 500 ms ITI) while the matching condition uses 5 trials per block (2800 ms stimulus, 400 ms ITI), with both block types lasting 18 seconds total. The contrast is relational vs matching.	Each run contains 6 task blocks (18 seconds each) plus 3 fixation blocks (16 seconds each), totaling approximately 2.6 minutes, with 2 runs making the entire relational processing task about 5.2 minutes long.
hcp	SOCIAL	Subjects are presented with short video clips of squares, circles, and triangles that either interact in some way or move randomly on the screen. Subjects must then judge whether the objects had an interaction that appears as if the shapes are taking into account each other's feelings and thoughts; if they are not sure, or if there is no obvious interaction between the shapes, and the movement appears random. The contrast is mental interactions vs random interactions.	Each run contains 5 video blocks (20 seconds each) plus 5 fixation blocks (15 seconds each), totaling approximately 2.9 minutes per run, with 2 runs making the entire social cognition task about 5.8 minutes long.
hcp	WM	Subjects view images from four categories (faces, places, tools, body parts) while performing either a low-demand task, where they match the current image to a cue (0-back task), or a high-demand task where they must indicate if a current image matches the one from 2 trials ago (2-back). The contrast is faces vs the average of all other categories (places, tools, body parts) across both 0-back and 2-back conditions.	Each run contains 8 task blocks (25 seconds each) plus 4 fixation blocks (15 seconds each), totaling approximately 4.3 minutes per run, with 2 runs making the entire working memory task about 8.6 minutes long.
hcp	REST	Eyes open with relaxed fixation on bright crosshair against dark background in darkened room. Phase encoding alternated between right-to-left (RL) and left-to-right (LR) within sessions.	2 sessions with 2 runs each session with 15 minutes per run. Total of 60 minutes with only 26 minutes used by the contributor for FC calculations.
pnc	REST	Exact quote: "During the resting-state scan, a fixation cross was displayed as images were acquired. Subjects were instructed to stay awake, keep their eyes open, fixate on the displayed crosshair, and remain still." (Satterthwaite et al. 2013)	6.18 minutes
slim	REST	Exact quote: "During the resting-state MRI scan, the subjects were instructed to lie down, close their eyes, and rest without thinking about any specific thing but to refrain from falling asleep." (Wei et al. 2018)	8 minutes

ukb	REST	Exact quote: "Subjects are instructed to keep their eyes fixated on a crosshair, relax and “think of nothing in particular” (Miller et al. 2018)	6.17 minutes

SI Table 2. Description of fMRI tasks and scan durations across all datasets. The experimental protocol, stimulus presentation details, and scan duration are provided for each dataset and task. Scan durations indicate total acquisition time and, where applicable, the portion used for functional connectivity (FC) calculations. Resting-state scans involved passive fixation with eyes open (ABCD, HBN, HCP, PNC, UKB) or closed (SLIM), ranging from 6-60 minutes. Task-based paradigms describe trial structure, timing parameters, and relevant contrasts used in analyses. Tasks included monetary incentive delay (MID), emotional n-back working memory (nBack), stop-signal inhibition (SST), emotion face matching (EMOTION), card gambling (GAMBLING), relational reasoning (RELATIONAL), social cognition with geometric animations (SOCIAL), and category-based working memory (WM). Task durations ranged from 2.2-8.6 minutes. [1, 2, 3, 4, 5, 6, 7, 8, 9, 10].

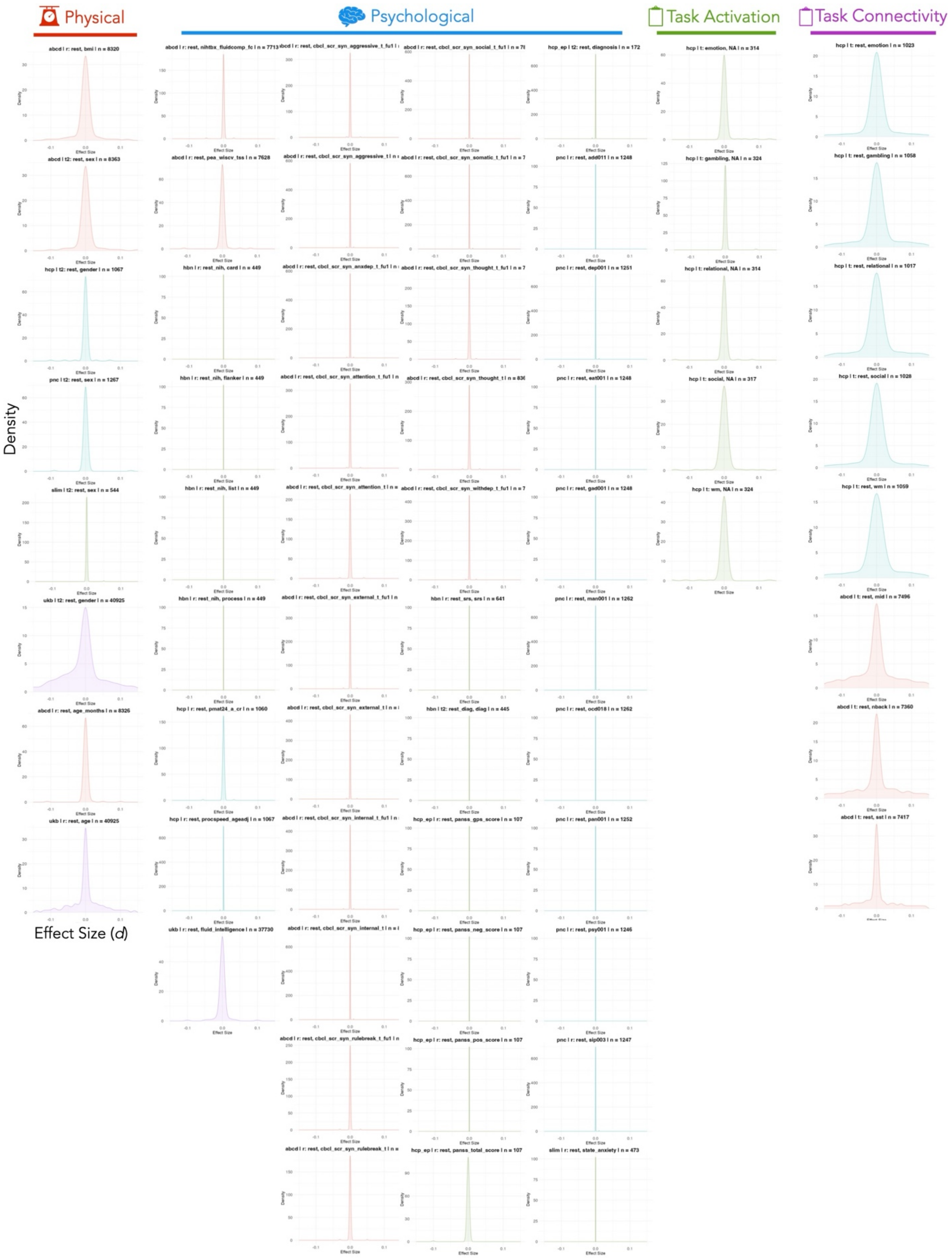

SI Figure 1. Uncorrected mass univariate cross-brain effect size distributions for all studies. Density plots show the distribution of Cohen’s d values across edges or voxels in each study.

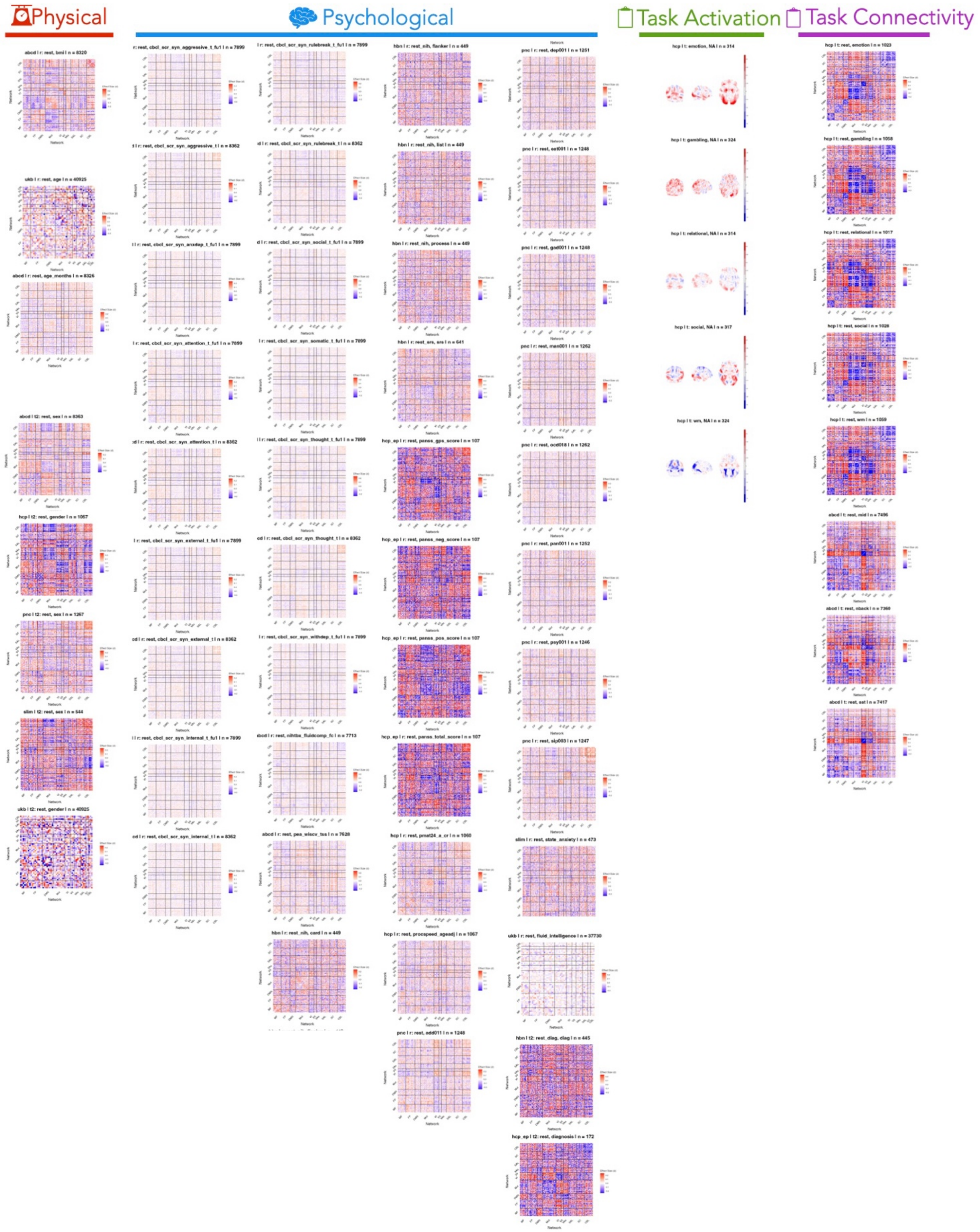

SI Figure 2. Spatial maps of effect size point estimates for all studies. Functional connectivity maps are arranged by network and activation maps are arranged spatially.

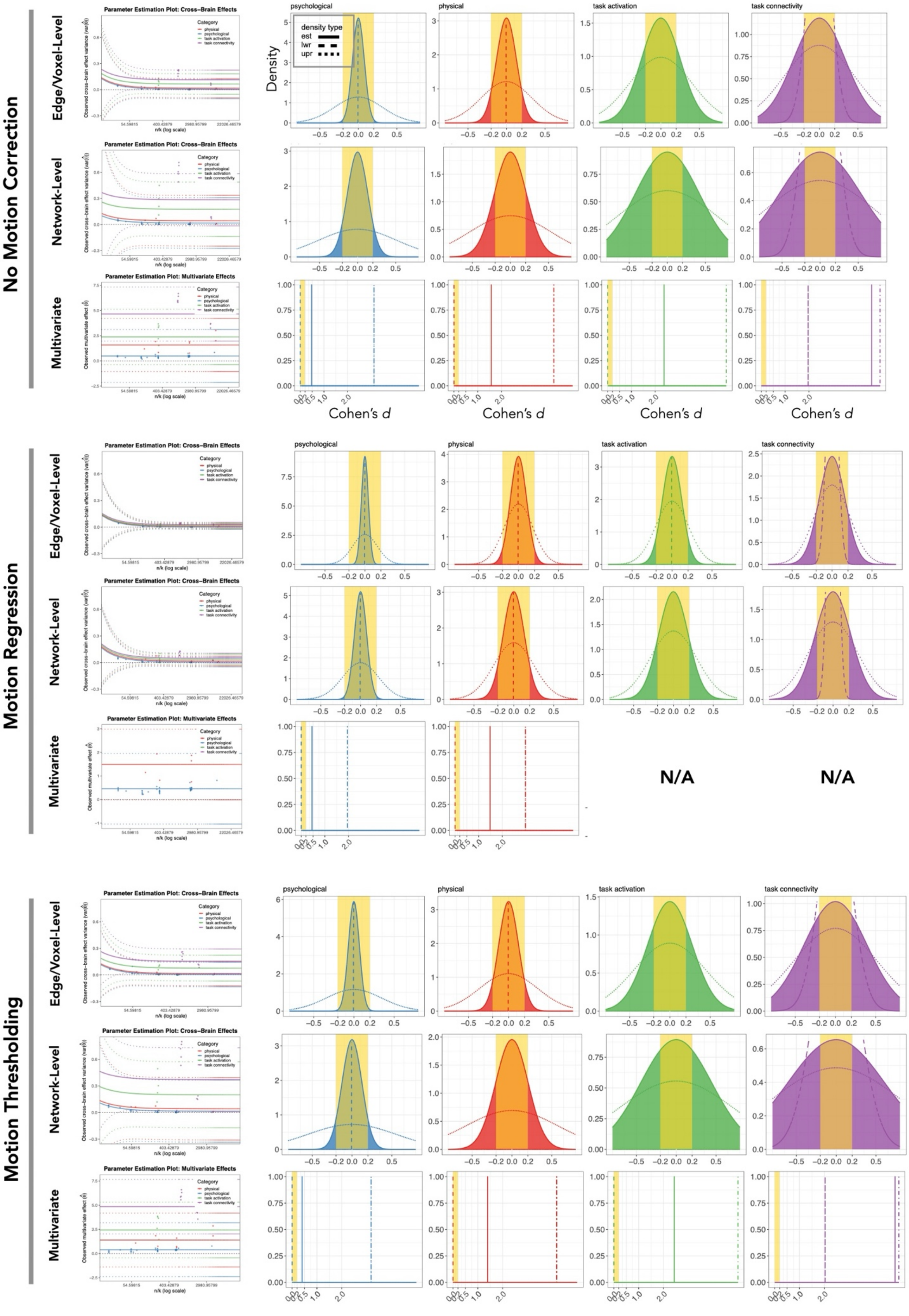

SI Figure 3. Cross-brain effect size distribution by motion deconfounding strategy. Parameter estimation plots and resulting corrected cross-brain effect size distributions for no control, motion regression, and motion thresholding.

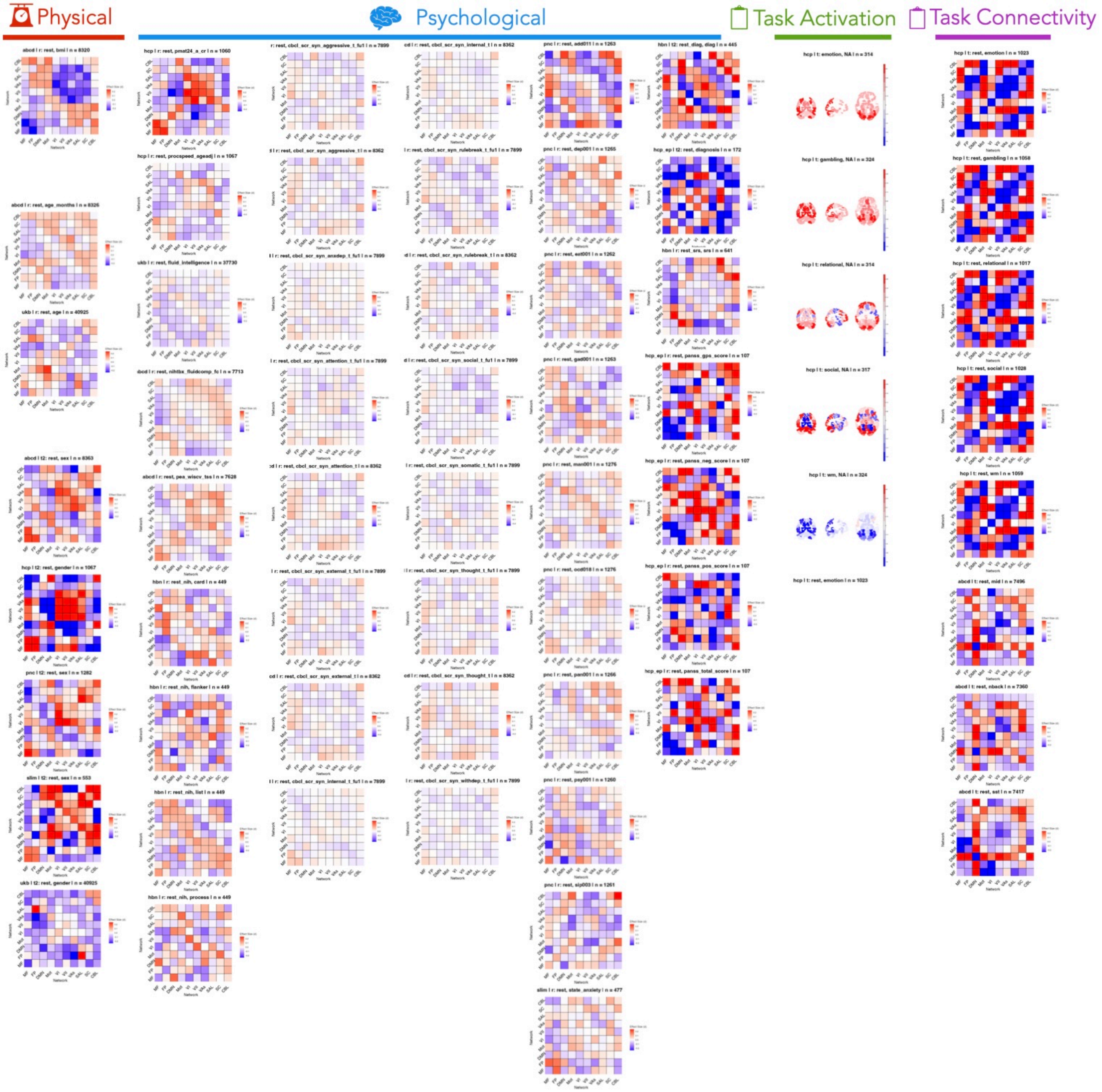

SI Figure 4. Spatial maps of network-level effect size point estimates for all studies. Functional connectivity is arranged by network, and activation maps are arranged spatially.

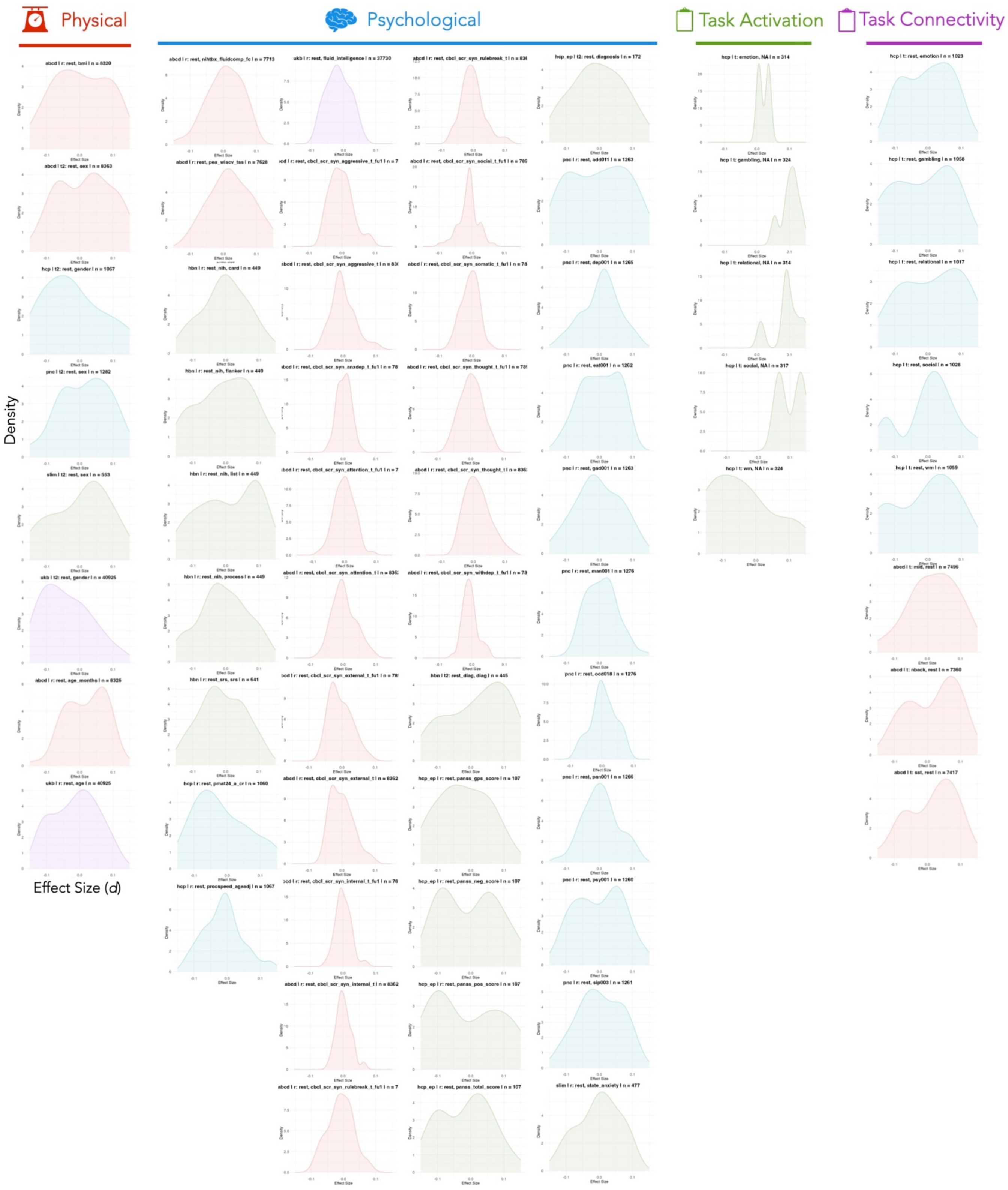

SI Figure 5. Network-level uncorrected mass univariate cross-brain effect size distributions for all studies. Density plots show the distribution of Cohen's d values across networks in each study.

	Edge-/voxel-level tau		
	lwr	*est*	*upr*
psychological	0	0.04319044	0.15521454
physical	0	0.10182351	0.1816839
task activation	0	0.12011973	0.20485957
task connectivity	0.06399325	0.16350695	0.22220241

	Network-level tau		
	lwr	*est*	*upr*
psychological	0	0.07698691	0.22552539
physical	0	0.13238866	0.25126588
task activation	0	0.1851588	0.2912742
task connectivity	0.05719886	0.2222323	0.30903508

	Multivariate point estimates		
	lwr	*est*	*upr*
psychological	-1.0406585	0.45715696	1.95497247
physical	-0.0129068	1.48490873	2.98272423
task connectivity	NA	NA	NA
task activation	NA	NA	NA

	Multivariate point estimates (0.1mm FFD thresholding)		
	lwr	*est*	*upr*
psychological	-2.3735835	0.40348583	3.18055514
physical	-1.3894623	1.39451864	4.17849958
task connectivity	2.02858739	4.84731202	7.66603665
task activation	-0.4381661	2.43596962	5.31010529

SI Table 3. Parameter estimates for effect size distributions: tau for edge-/voxel-level and network-level effect sizes, and point estimates for multivariate effect sizes.

	Edge-/voxel-level proportion		
	$|d|<0.1$	$|d|<0.2$	$|d|<0.5$
psychological	0.987	>0.999	1.000
physical	0.683	0.954	>0.999
task activation	0.595	0.904	>0.999
task connectivity	0.468	0.789	0.998

	Network-level proportion		
	$|d|<0.1$	$|d|<0.2$	$|d|<0.5$
psychological	0.789	0.988	1.000
physical	0.558	0.876	>0.999
task activation	0.401	0.707	0.992
task connectivity	0.351	0.637	0.977

	Multivariate results		
	|d|<0.1	*|d|<0.2*	*|d|<0.5*
psychological	0	0	1
physical	0	0	0
task connectivity	NA	NA	NA
task activation	NA	NA	NA

	Multivariate results (0.1mm FFD thresholding)		
	|d|<0.1	*|d|<0.2*	*|d|<0.5*
psychological	0	0	1
physical	0	0	0
task connectivity	0	0	0
task activation	0	0	0

SI Table 4. Proportion of effect sizes below conventional small and medium thresholds. For multivariate effects, this reflects whether the effect size point estimate is below the threshold (0=above, 1=below).

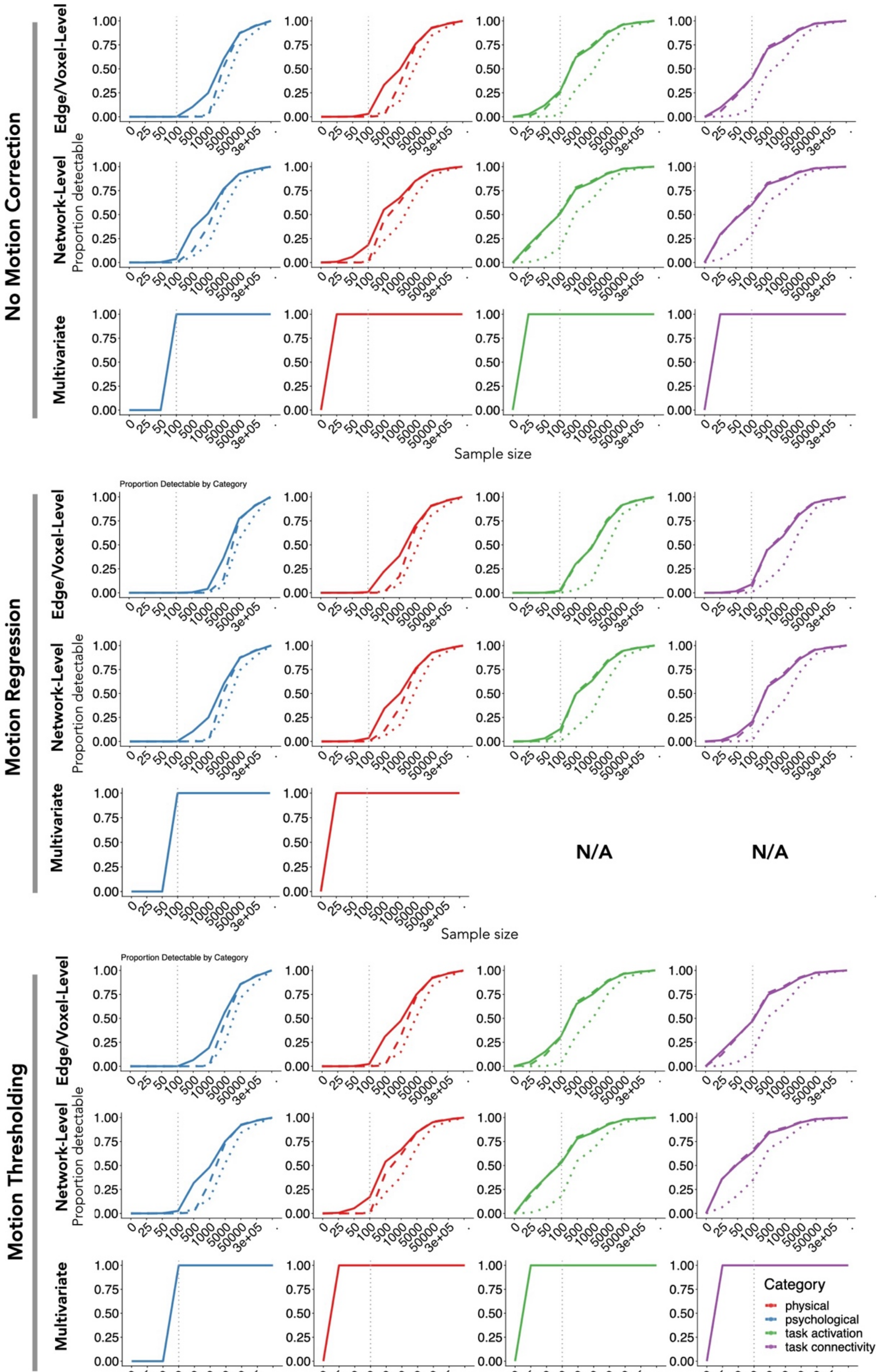

SI Figure 6. Power analysis results by motion deconfounding strategy. Sample size required to detect effects with 80% power for edge- or voxel-level (top), network-level (middle), or multivariate (bottom) effects for each outcome category (red and top, physical; blue and middle, psychological; green and bottom, task-based). Each plot shows the proportion of effects across the brain that can be detected with 80% power in each sample size category after Bonferroni, false discovery rate (FDR), or no correction.

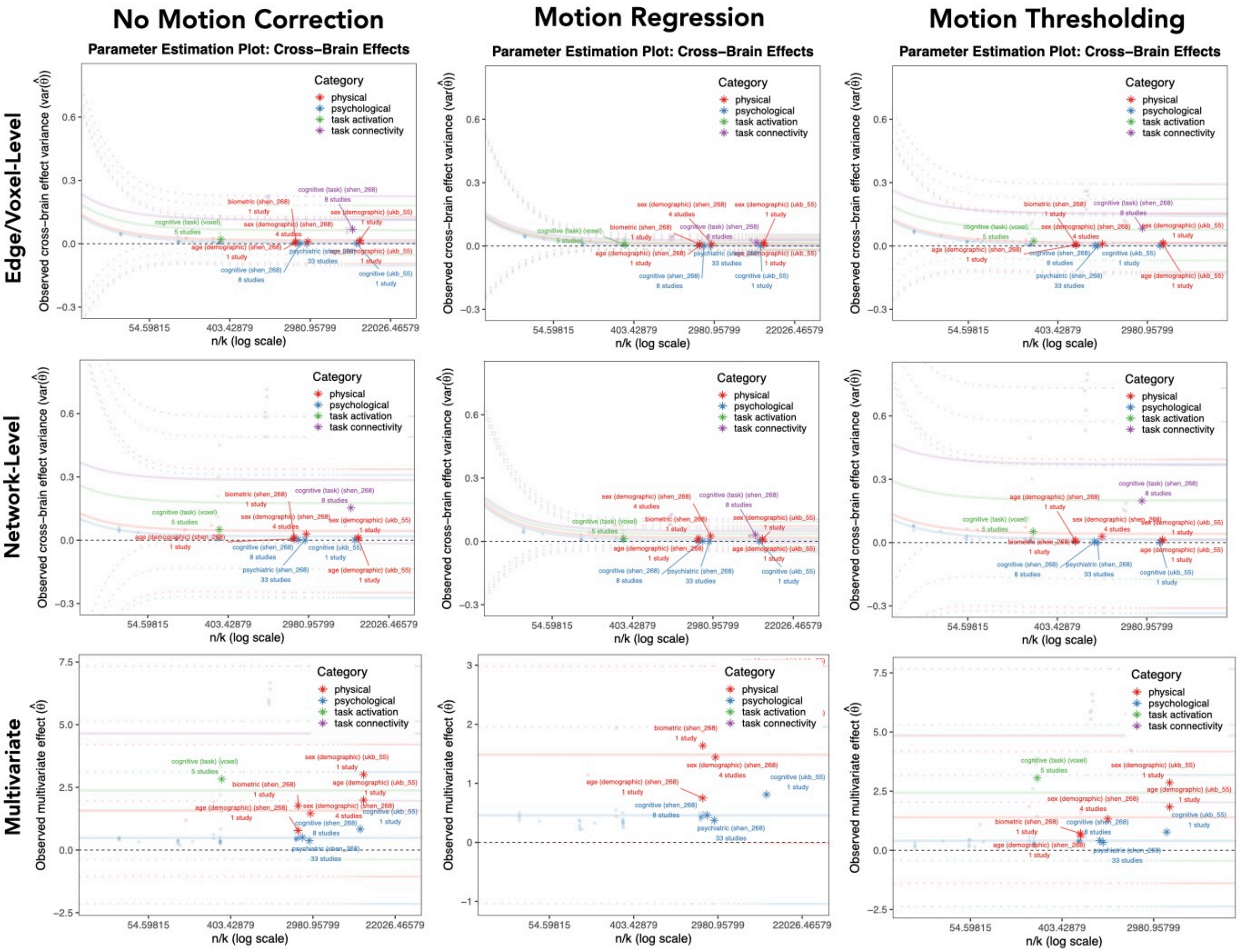

SI Figure 7. Meta-analyses from sub-categories by motion deconfounding procedure. τ_n^2 estimation plot (transparent) overlaid with $var(\hat{\theta})$ from meta-analysis results for each sub-category (stars).

Cohen's d	Sample size required for 80% power in within-subject design	Sample size *per group* required for 80% power in between-subject design
0.2	198	393
0.5	33	64
0.8	14	26

SI Table 5. Required sample size to detect univariate within vs. between-group effects at the commonly targeted 80% power level with a common alpha threshold of 0.05.

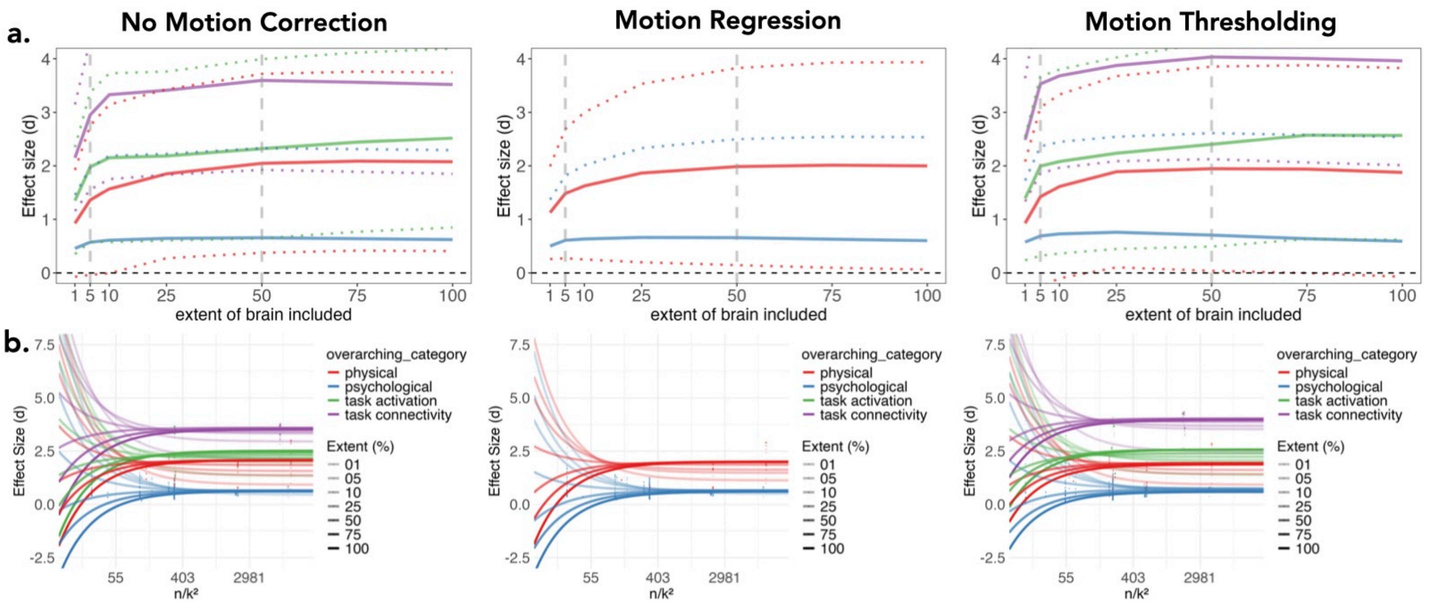

SI Figure 8. Effect size increases with the proportion of the brain included. Top panel: a) Multivariate effect size from the top *k* strongest effects, by outcome category (red, physical; blue, psychological; green, task activation; purple, task connectivity). The top 1%, 5%, 10%, 25%, 50%, 75%, and 100% marginal effects (x-axis) were selected and used to estimate the multivariate effect size. **b)**. The parameter estimation plot for each top *k* effect, with higher k in more opaque colors. Dotted lines denote top 5% and top 50% of effects.

References

1. Casey, B. J., Cannonier, T., Conley, M. I., Cohen, A. O., Barch, D. M., Heitzeg, M. M., ... & Dale, A. M. (2018). The adolescent brain cognitive development (ABCD) study: imaging acquisition across 21 sites. Developmental cognitive neuroscience, 32, 43-54.
2. Noble, S., Spann, M. N., Tokoglu, F., Shen, X., Constable, R. T., & Scheinost, D. (2017). Influences on the test–retest reliability of functional connectivity MRI and its relationship with behavioral utility. Cerebral cortex, 27(11), 5415-5429.
3. O'Connor, D., Potler, N. V., Kovacs, M., Xu, T., Ai, L., Pellman, J., ... & Milham, M. P. (2017). The Healthy Brain Network Serial Scanning Initiative: a resource for evaluating inter-individual differences and their reliabilities across scan conditions and sessions. Gigascience, 6(2), giw011.
4. Alexander, L. M., Escalera, J., Ai, L., Andreotti, C., Febre, K., Mangone, A., ... & Milham, M. P. (2017). An open resource for transdiagnostic research in pediatric mental health and learning disorders. Scientific data, 4(1), 1-26.
5. Barch, D. M., Burgess, G. C., Harms, M. P., Petersen, S. E., Schlaggar, B. L., Corbetta, M., ... & Van Essen, D. C. (2013). Function in the human connectome: task-fMRI and individual differences in behavior. Neuroimage, 80, 169-189.
6. Noble, S., Scheinost, D., & Constable, R. T. (2020). Cluster failure or power failure? Evaluating sensitivity in cluster-level inference. Neuroimage, 209, 116468.
7. Smith, S. M., Beckmann, C. F., Andersson, J., Auerbach, E. J., Bijsterbosch, J., Douaud, G., ... & WU-Minn HCP Consortium. (2013). Resting-state fMRI in the human connectome project. Neuroimage, 80, 144-168.
8. Satterthwaite, T. D., Elliott, M. A., Ruparel, K., Loughead, J., Prabhakaran, K., Calkins, M. E., ... & Gur, R. E. (2014). Neuroimaging of the Philadelphia Neurodevelopmental Cohort. NeuroImage, 86, 544-553.
9. Wei, D., Zhuang, K., Ai, L., Chen, Q., Yang, W., Liu, W., ... & Qiu, J. (2018). Structural and functional brain scans from the cross-sectional Southwest University adult lifespan dataset. Scientific data, 5(1), 1-10.
10. Miller, K. L., Alfaro-Almagro, F., Bangerter, N. K., Thomas, D. L., Yacoub, E., Xu, J., ... & Smith, S. M. (2016). Multimodal population brain imaging in the UK Biobank prospective epidemiological study. Nature neuroscience, 19(11), 1523-1536.
11. Jiang, R., Calhoun, V. D., Noble, S., Sui, J., Qi, S., Scheinost, D., ... & Zuo, N. (2023). A functional connectome signature of blood pressure in >30,000 participants from the UK Biobank. Cardiovascular Research, 119(6), 1427-1440.
